# Meiosis and Haploid Gametes in the Pathogen *Trypanosoma brucei*

**DOI:** 10.1016/j.cub.2013.11.044

**Published:** 2014-01-20

**Authors:** Lori Peacock, Mick Bailey, Mark Carrington, Wendy Gibson

**Affiliations:** 1 School of Biological Sciences, University of Bristol, Bristol BS8 1UG, UK; 2 Department of Clinical Veterinary Science, University of Bristol, Langford, Bristol BS40 5DU, UK; 3 Department of Biochemistry, University of Cambridge, Tennis Court Road, Cambridge CB2 1QW, UK

## Abstract

In eukaryote pathogens, sex is an important driving force in spreading genes for drug resistance, pathogenicity, and virulence [1]. For the parasitic trypanosomes that cause African sleeping sickness, mating occurs during transmission by the tsetse vector [2, 3] and involves meiosis [4], but haploid gametes have not yet been identified. Here, we show that meiosis is a normal part of development in the insect salivary glands for all subspecies of *Trypanosoma brucei*, including the human pathogens. By observing insect-derived trypanosomes during the window of peak expression of meiosis-specific genes, we identified promastigote-like (PL) cells that interacted with each other via their flagella and underwent fusion, as visualized by the mixing of cytoplasmic red and green fluorescent proteins. PL cells had a short, wide body, a very long anterior flagellum, and either one or two kinetoplasts, but only the anterior kinetoplast was associated with the flagellum. Measurement of nuclear DNA contents showed that PL cells were haploid relative to diploid metacyclics. Trypanosomes are among the earliest diverging eukaryotes, and our results support the hypothesis that meiosis and sexual reproduction are ubiquitous in eukaryotes and likely to have been early innovations [5].

## Highlights

• *Trypanosoma brucei* is a sexual organism
• Meiosis is a normal part of the trypanosome’s life cycle
• Identification of a novel haploid cell type with distinctive morphology
• First visualization of how trypanosomes mate

## Graphical Abstract

**Figure:**
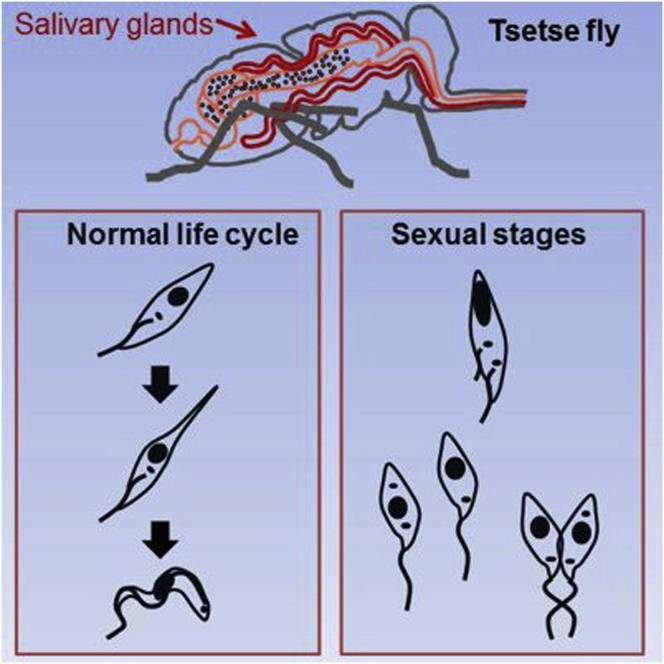

## Teaser

Peacock et al. use transgenic fluorescent cell lines to visualize sexual reproduction among trypanosomes from tsetse fly salivary glands, revealing the life-cycle stages involved in cell fusion.

## Results and Discussion

### Meiosis-Specific Genes Are Expressed in All Subspecies of *Trypanosoma brucei*

Meiosis-specific genes have been identified in several eukaryote pathogen genomes by phylogenomic analysis [5, 6], with demonstrated functionality in *Giardia intestinalis* [7] and *Trypanosoma brucei* [4]. Three functionally distinct, meiosis-specific proteins (MND1, DMC1, and HOP1) were expressed in *T. b. brucei* strain J10 [4], implying that meiosis might be an integral part of the trypanosome’s developmental cycle in the tsetse fly vector. To test this hypothesis and to obtain further information on the timing of meiosis, we extended the analysis to other strains and subspecies of *T. brucei*.

**Figure 1:**
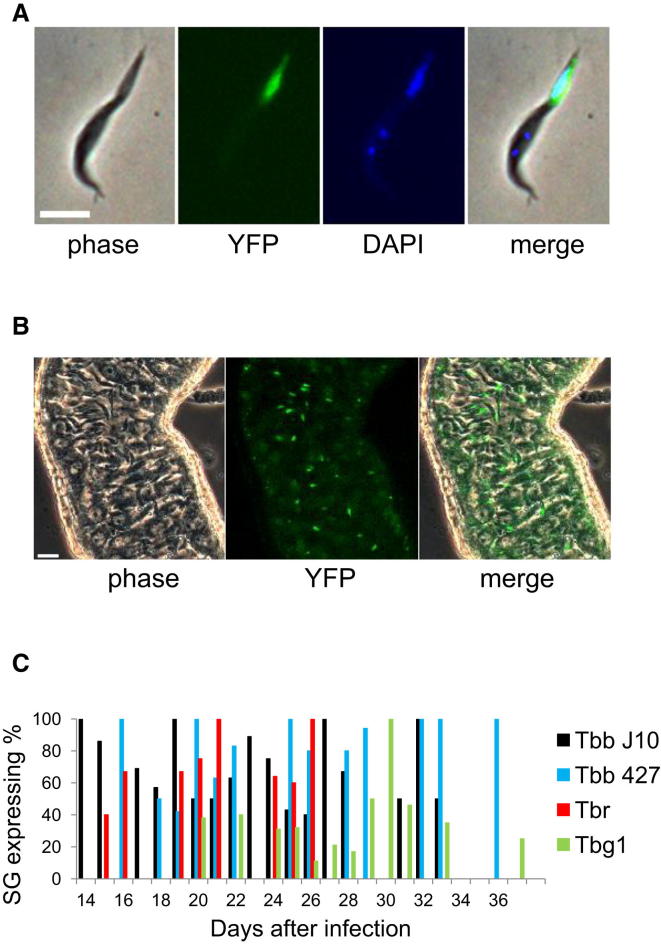
Expression of Meiosis-Specific YFP Fusion Proteins in Trypanosomes from Salivary Glands of Tsetse Flies (A) Expression of *YFP::DMC1* in *T. b. gambiense* DAL972. Scale bar, 5 μm. (B) Part of a tsetse salivary gland infected with *T. b. brucei* 427 var 3; live image. Many attached trypanosomes are expressing *YFP::HOP1*. Scale bar, 20 μm. (C) Aggregated results of expression of meiosis-specific genes over time. Flies were dissected from 14 to 38 days after infection and scored positive if the salivary glands contained a minimum of one trypanosome expressing a meiosis-specific gene (*MND1*, *DMC1*, or *HOP1*). The color denotes the trypanosome isolate.

The endogenous loci of three genes expressed solely during the prophase of meiosis I were modified so that each contained an N-terminal YFP tag (*YFP::MND1*, *YFP::DMC1*, *YFP::HOP1*). We used the following trypanosomes: *T. b. brucei* (Lister 427), *T. b. rhodesiense* (058), *T. b. gambiense* group 1 (DAL972), and *T. b. gambiense* group 2 (TH2), the last three being human pathogens. Each strain expressed the meiosis-specific genes exactly as previously observed for *T. b. brucei* J10 [4], confirming that the meiotic program takes place in the salivary glands (SG) during transmission of a clonal trypanosome strain (Figure 1). Although successful crosses of *T. b. brucei*, *T. b. rhodesiense*, and *T. b. gambiense* group 2 have been reported previously [8], this is the first indication that *T. b. gambiense* group 1 is capable of meiosis and, potentially, genetic exchange, despite lack of evidence of recombination in population genetics analyses [9, 10]. Sex provides the opportunity for new pathogen strains to arise by recombination, a phenomenon already suspected in *T. b. rhodesiense* [11], which has the capacity to become human infective by transfer of a single gene for human serum resistance (*SRA*) [12].

Trypanosomes expressing meiosis-specific genes were present in fly SG dissected between 14 and 38 days after infection (Figure 1C), with the highest frequency of expression around day 20. It follows that postmeiotic cells, including haploid gametes, should occur at the highest frequency at this time point. From measurements of nuclear DNA contents of different life-cycle stages, it has been assumed that *T. brucei* is diploid throughout its developmental cycle [13, 14, 15]. But mating in trypanosomes is believed to involve haploid nuclei or cells because the pattern of inheritance in experimental crosses is largely Mendelian [16]; moreover, triploid laboratory hybrids are often found, implying that fusion of haploid and diploid nuclei has taken place [3, 17, 18]. The expression of meiosis-specific genes preceded cell fusion [4], and hence meiosis generates the cells that subsequently undergo fusion. Our initial search for haploid trypanosomes by flow cytometry of SG-derived trypanosomes was unsuccessful because cell numbers were too low and amounts of fly tissue and debris too great to distinguish signal from noise; Movie S1 (available online) illustrates the suboptimal nature of the material under analysis. We therefore developed an alternative search strategy to find cells with gamete-like behavior in mixtures of SG-derived trypanosomes.

### Gamete-like Cell-Cell Interactions

**Figure 2:**
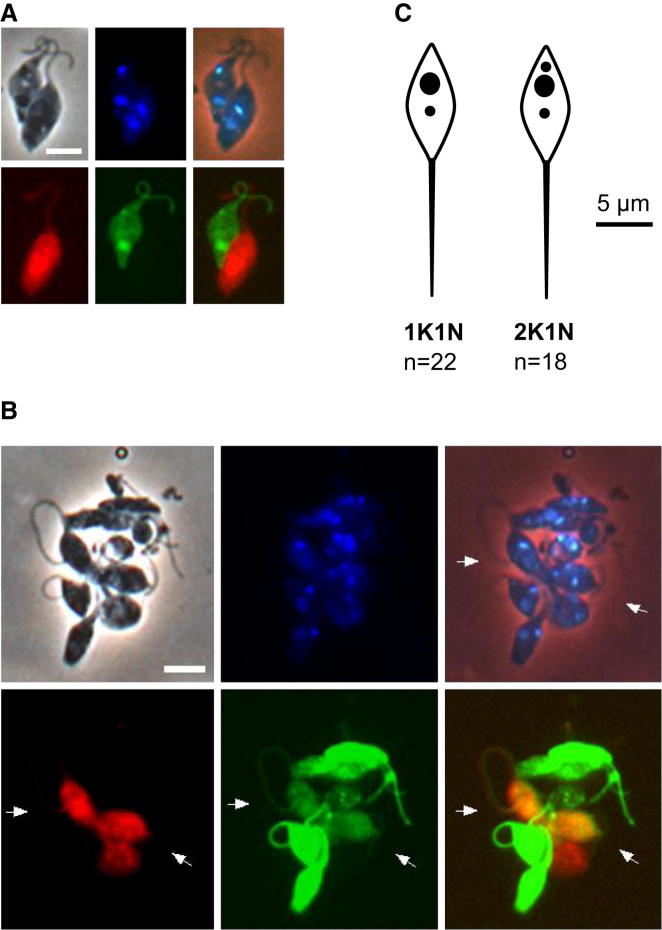
Interactions and Cytoplasmic Exchange between Salivary Gland-Derived Trypanosomes Ex Vivo Mix of trypanosome clones F1G1 (green fluorescence) and F1R1 (red fluorescence). (A) Pair of interacting trypanosomes; both are 2K1N promastigote-like (PL) cells; note the intertwined flagella and close proximity of cell bodies. Scale bar, 5 μm. (B) Cluster of mating trypanosomes; two trypanosomes (arrows) have exchanged cytoplasm, demonstrated by both green and red fluorescence. Scale bar, 10 μm. (C) Diagram compiled from measurements of 1K1N and 2K1N PL cells of 1738 GFP from salivary glands dissected 20 days after infection. See also Movies S2, S3, and S4.

Analysis of trypanosome crosses is facilitated by the incorporation of red fluorescent protein (RFP) or green fluorescent protein (GFP) into the parental clones, enabling hybrids to be identified by yellow fluorescence [3]. This system established that hybrid trypanosomes occur in the SG, not the midgut (MG), of infected flies and are found as early as 13 days after flies are coinfected with the parental clones [3]. Here, rather than carrying out the cross in vivo, we mixed the parental clones in vitro using trypanosomes derived from the SG of flies separately infected with either red or green fluorescent cell lines. Trypanosomes were harvested from fly SG during the window of peak expression of meiosis-specific genes, and the ex vivo mixtures were observed as living cells in microslides over the course of about an hour, and also after fixation on microscope slides. Twenty replicate experiments were carried out using mating-compatible pairs J10 RFP/1738 GFP or F1G2/F1R1 (Table S1A). Small clusters of two or more red and green fluorescent trypanosomes, as well as clusters of single color trypanosomes, were observed within 10 min of mixing the red and green fluorescent SG-derived parental cells (Figure 2; Movies S2 and S3).

We focused the search for gamete interactions on the red-green clusters because single-color clusters might also arise from cell division. All red-green clusters contained at least one trypanosome with a short, wide body and long anterior flagellum. These trypanosomes were observed to interact by intertwining their long flagella, often drawing the cell bodies into close proximity (Figure 2A). These interactions are reminiscent of the recognition and attachment of gametes of the unicellular plant *Chlamydomonas reinhardtii* via their paired flagella [19]. In the clusters, the trypanosomes were highly active, frequently changing position (Movies S2 and S3). These distinctive cell interactions had not been observed previously in our analysis of experimental crosses [3, 18, 20]. To rule out the possibility that the SG cells were “sticky,” we set up mixtures of MG- and SG-derived trypanosomes in all possible combinations (Table S1B). The distinctive flagellar interactions were observed only among SG-derived trypanosomes and did not occur in mixtures of SG- and MG-derived trypanosomes or between MG-derived trypanosomes; when red and green trypanosomes were found together in these mixtures, they were associated with debris or dead trypanosomes.

Yellow fluorescent trypanosomes started to appear within 30 min of mixing the SG-derived parental lines (Figure 2B) and were observed in 11 of 20 experiments (Table S1A). The rapid appearance of yellow fluorescent trypanosomes signifies fusion of cell membranes and exchange of cytoplasm between red and green fluorescent cells rather than de novo synthesis of fluorescent proteins, which would take several hours. These results demonstrate that clonal trypanosome populations derived from the SG already contain fusion-competent cells, ruling out the hypothesis that these cells are generated after recognition of nonself among trypanosomes of different genotypes [21].

### Morphology of Gamete-like Trypanosomes

The trypanosomes implicated as trypanosome gametes had a distinctive morphology, and DAPI staining revealed either one kinetoplast and one nucleus (1K1N) or two kinetoplasts and one nucleus (2K1N) (Figure 2; Movies S3 and S4). In trypanosome biology, there is no term for a cell with this conformation. The closest is promastigote, which refers to a cell with an antenuclear kinetoplast and a flagellum that emerges anteriorly without connection to the cell body via an undulating membrane [22]. In the following, we refer to these 1K1N and 2K1N cells collectively as promastigote-like (PL) cells. Both 1K1N and 2K1N PL cells displayed gamete-like cell-cell interactions, but we were unable to distinguish different behavior during mating.

**Figure 3:**
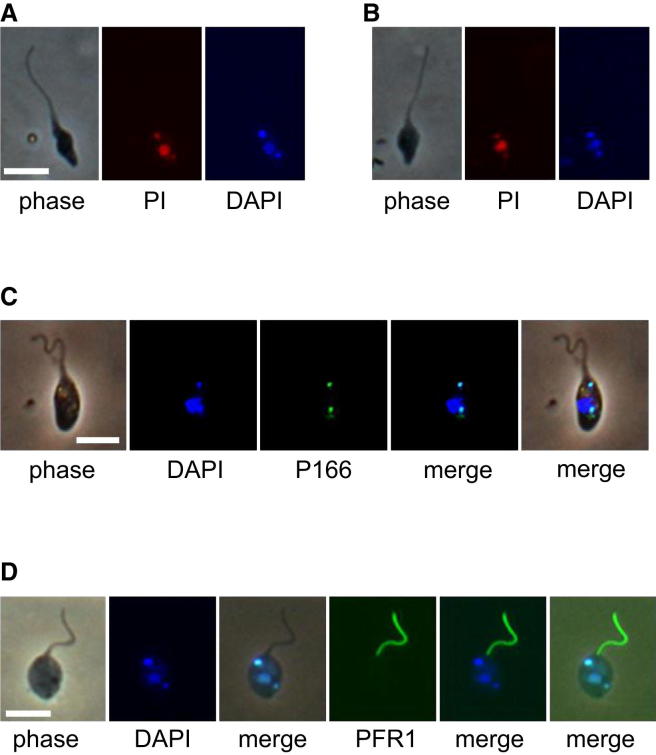
Morphology of 2K1N Promastigote-like Trypanosomes (A and B) Differential staining of DNA with DAPI and PI. DAPI preferentially binds to AT-rich DNA compared to PI, allowing the AT-rich kinetoplast DNA to be distinguished from nuclear DNA. Two different 2K1N PL cells are shown. (C) Localization of protein P166 from tripartite attachment complex in 2K1N PL cell of *T. b. brucei* J10 carrying fusion construct *YFP::P166*. (D) Visualization of flagellum in 2K1N PL cell of *T. b. brucei* J10 carrying fusion construct *YFP::PFR1*. The trypanosome has a single flagellum associated with the anterior kinetoplast. Scale bars, 10 μm. See also Figure S1.

The presence of two kinetoplasts in the 2K1N PL cells was confirmed by differential staining with DAPI and propidium iodide (PI) [23] (Figures 3A and 3B) and also by visualization of the subcellular location of a *YFP::P166* fusion construct (Figures 3C and S1A). P166 is an intrinsic component of the tripartite attachment complex (TAC), the filamentous structure that physically attaches the kinetoplast to the basal body of the flagellum [24]. The intensity of fluorescence of P166 in the anterior TAC was usually greater than that in the posterior TAC (Figure S1A), perhaps associated with the presence of a flagellum attached to the anterior kinetoplast.

The 2K1N PL cells had only one flagellum, although the two kinetoplasts were widely separated at the two poles of the cell on either side of the nucleus, and a new flagellum would normally be evident at this stage of cell division. To detect whether a second flagellum was present but not discernible by light microscopy, we visualized the subcellular localization of PFR1, a major structural protein of the paraflagellar rod (PFR) [25], using cells expressing the fusion *YFP::PFR1* (Figures 3D and S1B). Of 26 2K1N PL cells examined, 23 (88%) had only a single fluorescent flagellum, arising near the anterior kinetoplast. The remaining three cells had two flagella, the posterior flagellum appearing as a tiny dot of fluorescence near the posterior kinetoplast (Figure S1B). The PFR extends from the point at which the flagellum emerges from the flagellar pocket to its distal tip, and thus PFR1 is absent from a transitional zone of the flagellum adjacent to the basal body. We therefore cannot rule out the presence in all 2K1N cells of a very short flagellum that does not extend beyond the flagellar pocket, as seen in *Leishmania* amastigotes [26].

### Measurement of DNA Contents

**Figure 4:**
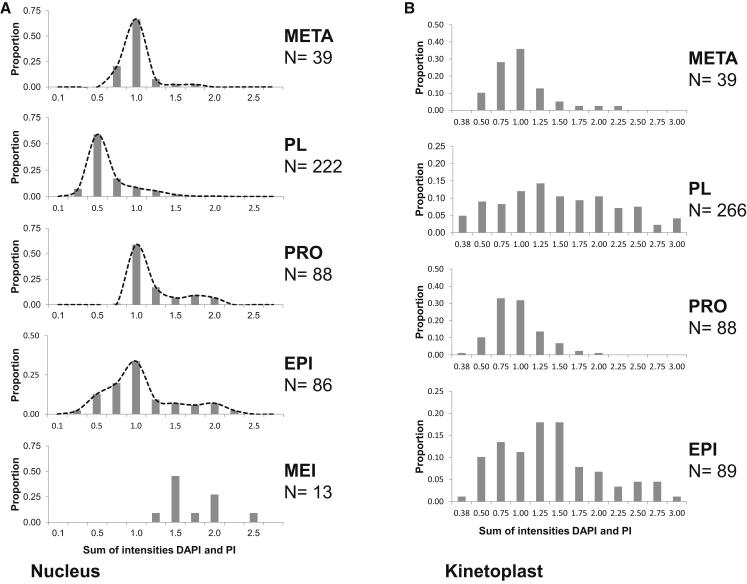
Nuclear and Kinetoplast DNA Contents of Salivary Gland-Derived Trypanosomes Trypanosomes (1738 GFP) from flies dissected 17–20 days after infection were fixed and stained with DAPI and PI before imaging under uniform conditions. Total pixel intensities were measured for both DAPI and PI-stained nuclei and kinetoplasts using ImageJ (http://rsb.info.nih.gov/ij) and normalized relative to G1-arrested metacyclics (META, nuclear DNA content = 1.0; kinetoplast DNA content = 1.0). Other cells were categorized by morphology into promastigote-like cells (PL; see Figure 2C), procyclics (PRO; long trypomastigote with round or oval nucleus), epimastigotes (EPI; nucleus posterior to kinetoplast), cells in meiosis I (MEI; epimastigote with posterior nucleus, two anterior kinetoplasts and associated flagella). N denotes number of organelles measured in each cell category. (A) Nuclear DNA contents. Each histogram shows the frequency distribution of total normalized pixel intensities per nucleus; values on the x axis are the median for each bin (bin size = 0.25). The superimposed dotted line is a smooth curve. (B) Kinetoplast DNA contents. Each histogram shows the frequency distribution of total normalized pixel intensities per kinetoplast; values on the x axis are the median for each bin (bin size = 0.25).

The above evidence on interactions and cytoplasmic fusion implicates PL cells as trypanosome gametes; the crucial question is whether they are haploid, a defining feature of eukaryote gametes. PL cells were present in small numbers in live preparations of SG spillout (Movies S1 and S5), together with metacyclics and other unattached trypanosomes. Fixed cells were stained with both DAPI and propidium iodide (PI) before measurement of total fluorescence intensity of the nucleus. This provided two independent measures of nuclear DNA content, because DAPI and PI have different DNA binding characteristics [23] (although DNA binding could in principle be affected by chromatin packing). Both 2K1N and 1K1N PL cells were haploid relative to metacyclics, the diploid, G1-arrested, mammal-infective stage also present in the SG [27] (Figure 4A). The combined DAPI and PI pixel intensities of PL cells peaked at 0.5, compared to metacyclics at 1.0 (2C DNA content) (Figure 4A).

Procyclics from the fly midgut had a major peak at 1.0 and a smaller peak at around 2.0, consistent with the 2C and 4C peaks expected for this proliferating cell population (Figure 4A). Epimastigotes are also a proliferative insect stage, which are characteristically found attached to the SG epithelium via the flagellum, but here we examined free epimastigotes in the SG spillout; these cells did not have the elongated posterior “nozzle” typical of attached epimastigotes. The population shows nuclear DNA content peaks at 1.0 (2C) and 2.0 (4C) like procyclics, but there is a noticeable shoulder of nuclei with DNA content <1.0 (Figure 4A). Reexamination of this population revealed ten cells apparently in division, as each had two nuclei (either 3K2N or 2K2N), with nuclear DNA contents in the haploid range. We speculate that some of these are cells in reduction division (meiosis II), but they were difficult to distinguish from dividing PL cells, except by the extreme length of the flagellum (Figure S2). A total of 12 dividing PL cells was recorded among ∼600 cells examined; because this was a clonal trypanosome population, this cell morphology is unlikely to arise by fusion of PL cells. A small number of epimastigotes in meiosis I was identified by morphology (2K epimastigote with posterior nucleus and two short flagella; Figure S1B); these had nuclear DNA contents consistent with 4C or greater (MEI, Figure 4). Meiotic cells are often found attached inside the SG (Figure 1B), explaining the small number of free meiotic cells observed.

PL cells frequently had a large, elongated anterior kinetoplast, which was highly conspicuous in DAPI staining (Figure 2), confirmed by measurement of kinetoplast DNA (kDNA) contents relative to the unit kinetoplast of metacyclics (Figure 4B). Twice the unit amount of kDNA usually indicates a kinetoplast about to divide, suggesting a simple hypothesis for the generation of 2K1N PL cells by kinetoplast division in 1K1N PL cells. However, this is contradicted by the fact that both 1K1N and 2K1N PL cells had enlarged kinetoplasts. Alternatively, 1K1N and 2K1N PL cells might arise by unequal division of a 3K1N cell, because both PL cell types were found in approximately equal numbers. Both 3K1N and 3K2N cells were observed (e.g., Figure S2E).

In summary, we have shown that meiosis is a normal part of the developmental cycle of *T. brucei* in the tsetse fly and have identified a novel haploid PL cell that displays the behavior expected of a gamete. When PL cells of different strains are mixed, they readily form pairs or clusters and undergo cytoplasmic fusion. We conclude that the haploid PL cells are trypanosome gametes, although formal proof will require the demonstration of exchange of nuclear and kinetoplast DNA. Until now, the extent and significance of sex in kinetoplastid parasites have been controversial [28], but this study reveals that *T. brucei* is essentially a sexual organism. Trypanosomes belong to supergroup Excavata and are among the earliest diverging eukaryotes [29]. Hence, these results support the hypothesis that meiosis and sexual reproduction are ancestral and ubiquitous features of eukaryotes [5].

## Experimental Procedures

### Trypanosomes

The following tsetse-transmissible strains of *Trypanosoma brucei* subspecies were used: *T. b. brucei* J10 (MCRO/ZM/73/J10 CLONE 1), 1738 (MOVS/KE/70/EATRO 1738), Lister 427 (MOVS/UG/60/427 VARIANT 3); *T. b. rhodesiense* 058 (MHOM/ZM/74/058 CLONE B); *T. b. gambiense* group 1 DAL972 (MHOM/CI/86/DAL972 CLONE 1); *T. b. gambiense* group 2 (MHOM/CI/78/TH2). The experimental cross of J10 and 1738 carrying cytoplasmically expressed genes for fluorescent proteins is described in [3] and produced hybrid progeny F1G2 and F1R1. Procyclic form (PF) trypanosomes were grown in Cunningham’s medium (CM) [30] supplemented with 10% v/v heat-inactivated fetal calf serum, 5 μg/ml hemin, and 10 μg/ml gentamycin at 27°C. Tsetse flies were infected with trypanosomes and dissected essentially as described previously [4].

### Transfection

Fusion constructs of the *YFP* gene with the homologs of meiosis-specific genes (*DMC1*, *Tb09.211.1210*; *HOP1*, *Tb10.70.1530*; *MND1*, *Tb11.02.3380*) and *PFR1* (*Tb927.3.4290*) were described in [4]. P166, a component of the tripartite attachment complex connecting the kinetoplast and basal body of the flagellum, was also tagged with YFP (accession number FJ407182) [24]. PFs were transfected by electroporation, antibiotic selected, and cloned as previously described [4].

### Microscopy

For analysis of expression of fusion proteins associated with meiosis, fly organs (SG and alimentary tract from the proventriculus to the hindgut) were dissected in a drop of PBS and examined for the presence of fluorescent trypanosomes using a DMRB microscope (Leica) equipped with a Retiga Exi camera (QImaging) and Volocity software (PerkinElmer). Cells were fixed in 2% w/v paraformaldehyde (PFA) at room temperature for 20 min and stained with DAPI in VECTASHIELD mounting medium (Vector Laboratories) to visualize the nucleus and kinetoplast. For analysis of mating between red and green fluorescent trypanosomes, SGs were dissected directly into CM. Medium containing spilled-out trypanosomes was introduced into a microslide capillary for live imaging as above, together with Hoechst live stain if required. Alternatively, preparations were spread on microscope slides using a cytospin after fixation in 2% w/v PFA and were DAPI stained and mounted as above. Methodology for measurement of DNA content is fully described in the Supplemental Experimental Procedures.

## Supplemental Information

Document S1. Supplemental Experimental Procedures, Figures S1 and S2, and Table S1

Movie S1. Trypanosomes and Debris Spilling Out from a Dissected Salivary Gland, Related to Experimental Procedures Two promastigote-like (PL) cells (arrows); phase-contrast sequence.

Movie S2. Gamete-like Cell-Cell Interactions, Related to Results Red PL cell interacting with green PL cell from salivary glands dissected at 21 days. The two trypanosomes are interacting by intertwining their long flagella, and the cell bodies are also in close contact. The red fluorescent trypanosome appears to be dividing: it has two flagella and a cleavage furrow. Shown is a phase-contrast sequence followed by triple-pass UV filter to visualize green and red fluorescence simultaneously, followed by a further phase-contrast sequence and ending with separate red and green fluorescence images in sequence.

Movie S3. Gamete-like Cell-Cell Interactions, Related to Results Red PL cell interacting with green PL cell from salivary glands dissected at 19 days. The two trypanosomes are interacting via their flagella. Phase-contrast sequence is followed by brief visualization of green followed by red fluorescence, followed by prolonged visualization of nuclei and kinetoplasts by Hoechst fluorescence; during this part of the sequence, the upper trypanosome appears to be 1K1N and the lower 2K1N. The subsequent phase-contrast sequence ends with separate red and green fluorescence images in sequence; the two trypanosomes remain as separate cells and do not exchange cytoplasm.

Movie S4. 2K1N PL Cell, Related to Results Phase-contrast sequence followed by Hoechst fluorescence to visualize nucleus and kinetoplasts, ending with visualization of YFP::PFR in the flagellum. PL cell of *T. brucei* J10 HOP YPFR from salivary glands dissected at 19 days.

Movie S5. Salivary Gland Material used for Measurement of DNA Contents, Related to Results Spillout from a dissected salivary gland showing PL cell beside the salivary gland (bottom left). Phase-contrast sequence.
