# Supplementary material for: Meiosis and Haploid Gametes in the Pathogen Trypanosoma brucei
Source: Curr Biol. 2014 Jan 20;24(2):181–6. doi: 10.1016/j.cub.2013.11.044 (PMC3928991; doi:10.1016/j.cub.2013.11.044)
Supplement: Document S1. Supplemental Experimental Procedures, Figures S1 and S2, and Table S1 [file mmc1.pdf]

Current Biology, Volume *24*

Meiosis and Haploid Gametes in the Pathogen *Trypanosoma brucei*

Lori Peacock, Mick Bailey, Mark Carrington, and Wendy Gibson

Inventory of Supplemental Information

Figures
Figure S1 related to Figure 3. Shows additional images.
Figure S2 related to Figure 4. Shows additional images.

Tables
Table 1 related to Figure 2. Shows the body of experimental data from which these images were chosen.

Supplemental Experimental Procedures
Gives detailed protocol for measurement of DNA contents, together with ImageJ macros used to analyse the data.

Supplemental References
References from Supplemental Experimental Procedures.

Supplemental Information

Supplemental Data

Figure S1A

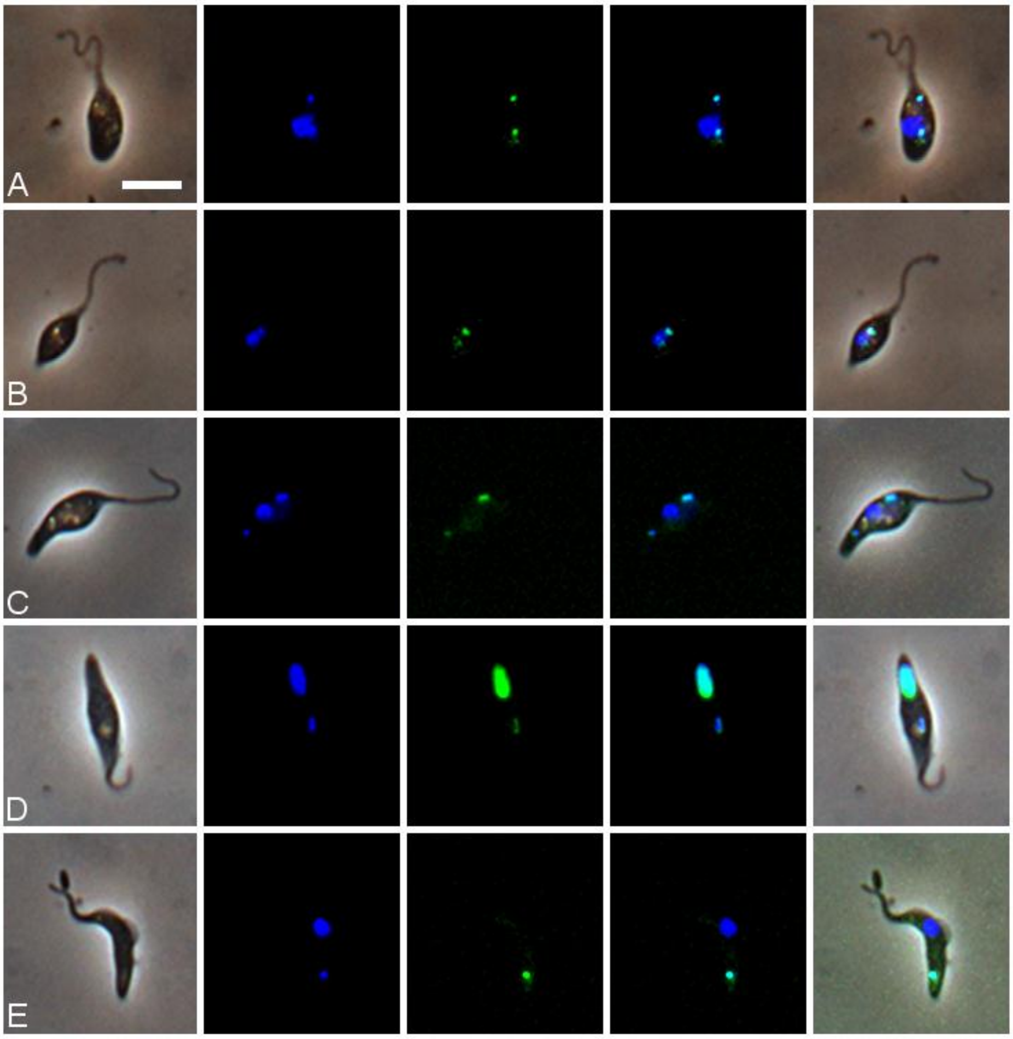

Figure S1B

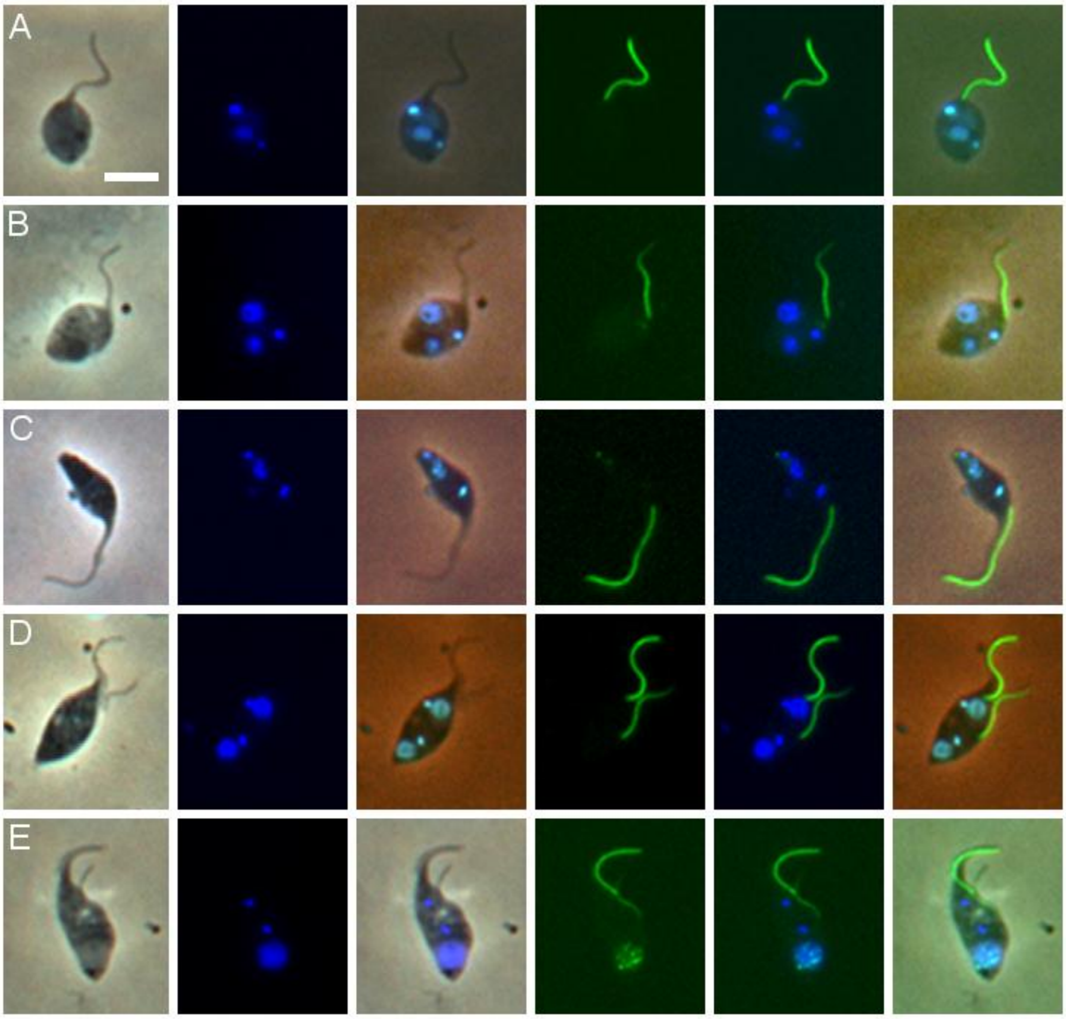

Figure S2

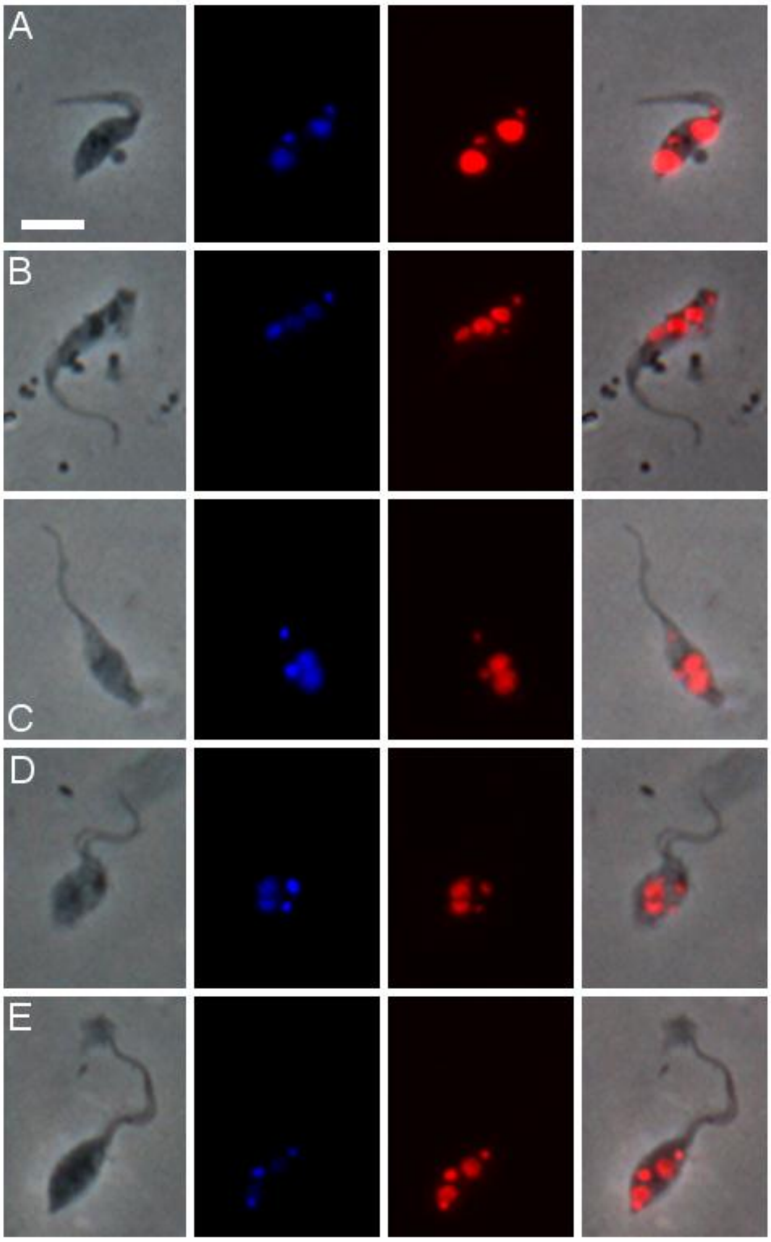

Figure legends

Figure S1. Related to Figure 3

Morphology of PL cells. **A.** Kinetoplast. Localisation of protein P166 from tripartite attachment complex in J10 MND P166 derived from tsetse salivary gland. Each row shows L to R: phase contrast, DAPI, yellow fluorescence, merge of DAPI and yellow fluorescence, merge with phase contrast image. **A-C**. 2K1N PL cells. **D**. 2K1N meiotic cell expressing *YFP::MND1* in the nucleus as well as *YFP::P166.* **E**. 1K1N metacyclic trypomastigote. Scale bar = 10 µm. **B.** Flagellum. Salivary gland derived trypanosomes of *T. b. brucei* J10 carrying fusion constructs *YFP::DMC1* and *YFP::PFR1*. Each row L to R: phase contrast, DAPI, merge with phase contrast image, yellow fluorescence, merge of DAPI and yellow fluorescence, merge of all images. **A-C**. 2K1N PL cells; cells A and B have a single flagellum associated with the anterior kinetoplast; cell C also has a small dot of fluorescence adjacent to the posterior kinetoplast. **D**. 2K2N dividing cell. **E**. 2K1N meiotic cell expressing *YFP::DMC1* in the nucleus as well as *YFP::PFR.* Scale bar = 10 µm.

Figure S2. Related to Figure 4

Dividing cells with haploid nuclei. L to R, phase contrast, DAPI fluorescence, PI fluorescence, merge. **A**. 2K2N epimastigote with nuclear DNA contents in diploid range. **B**. 2K2N epimastigote with nuclear DNA contents in haploid range. **C**. 2K2N PL cell. **D**. 2K2N dividing PL cell. **E**. 3K2N PL cell with a flagellum that appears thicker than usual and may be two flagella running together. Scale bar = 5 µm.

Table 1 Related to Figure 2

A: *Ex vivo* crosses of salivary gland-derived trypanosomes.

Cross		No. of experiments		
		Total	Red-Green clusters	Yellow trypanosomes
J10RFP	1738GFP	15	11 (73%)	6 (40%)
F1R1	F1G2	5	5 (100%)	5 (100%)
Total		20	16 (80%)	11 (55%)

B: Pairwise mixing of salivary gland (SG) and midgut (MG) derived trypanosomes from J10RFP and 1738GFP. All preparations were from the same batches of infected flies.

Mixture		Trypanosome clusters			No. of red-green clusters with yellow trypanosomes
J10RFP	1738GFP	Red-Red	Green-Green	Red-Green	
SG	SG	7 (8%)	46 (53%)	34 (39%)	3 (9%)
MG	SG	10 (14%)	47 (66%)	14 (20%)	0
SG	MG	9 (38%)	7 (29%)	8 (33%)	0
MG	MG	15 (40%)	7 (20%)	15 (40%)	0

Supplemental Experimental Procedures

Measurement of DNA contents

Published methods for measurement of trypanosome nuclear and kinetoplast DNA contents after DAPI [S1] or DAPI/PI (propidium iodide) [S2] staining were adapted for the small numbers of cells available from infected salivary glands (SG) at early timepoints (15 to 23 days after infection). SG from 10-15 flies were pooled in 50 µl of Cunningham's medium (CM) and briefly agitated. CM containing trypanosomes spilt out from the SG was carefully removed and placed in a clean tube. Cells were fixed in 2% w/v paraformaldehyde (PFA) at 4°C for 20 minutes, followed by permeabilization with 0.5% w/v Triton (final concentration) for 7 minutes at 4°C. Cells were washed twice in phosphate buffered saline (PBS) before RNAse incubation (50 µg/ml final concentration; 30 minutes at room temperature) followed by PI staining (4 µg/ml final concentration; 30 minutes at room temperature in the dark). The volume was brought to 200 µl and cells spread in a circle of 5 mm diameter on a microscope slide using a Shandon Cytospin 4 (1350 rpm, 5 minutes room temperature). Preparations were stained with DAPI in VECTASHIELD mounting medium (Vector Laboratories) and viewed immediately using a DMRB microscope (Leica) equipped with a Retiga Exi camera (QImaging) and Volocity software (PerkinElmer). The cytospin circle was scanned systematically from top to bottom by phase contrast at 400x, capturing each trypanosome image using a standard set of exposure settings for PI, DAPI and phase contrast, in that order. Care was taken not to expose the field of view to UV light more than once to avoid photobleaching.

ImageJ (http://rsb.info.nih.gov/ij) was used to analyse the digital images of fixed, DAPI and PI stained trypanosomes. For each set of images, trypanosomes were first identified by morphology in the phase contrast image; typically only one or two trypanosomes were present. The corresponding 48 bit PI and DAPI images were then quantitated as follows. Each image was split into 16 bit blue, green and red colour channels; for PI, the red image was quantitated, and for DAPI the blue image. The background was subtracted using a rolling ball radius of 10 µm. The image was then duplicated to create a

thresholded mask with outlines of the nucleus and kinetoplast (particle analysis); the result was inspected and manually redrawn if the nucleus and kinetoplast were too close for resolution as separate objects; images where the kinetoplast overlay the nucleus were rejected. The intensity of each pixel in each object was then summed to yield the raw intensity density (total pixel intensity). Macros were written to automate this process for DAPI and PI images (see below). The cell preparation, staining and image analysis methodology were refined by initial experiments on culture and SG-derived trypanosomes.

To obtain sufficient cells for analysis, results from six separate experiments were combined, each consisting of images derived from a single cytospin of SG-derived trypanosomes of *T. b. brucei* 1738 GFP dissected on days 17 - 20 after infection. Cells were identified morphologically as metacyclic (short trypomastigote 1K1N), procyclic (long trypomastigote with round or oval nucleus), meiotic (epimastigote with posterior nucleus, two anterior kinetoplasts and associated flagella), epimastigote (nucleus posterior to kinetoplast), promastigote-like (elongated flagellum), or unclassified. For each cell, total pixel intensities for each organelle were normalised relative to the mean organelle DNA contents of metacyclics (= 1.0) in that experiment. The normalised values for each cell type were then plotted as a frequency distribution (bin size 0.25) using DAPI, PI or mean DAPI and PI values. Combining the DAPI and PI results reduced the error in our measurements by approximately half as judged by the variance of DNA contents for metacyclics: variance DAPI = 0.0694; variance PI = 0.0621; variance mean DAPI and PI = 0.0360.

ImageJ macros

NB. Set scale appropriate to microscope system; in our setup 6.236 pixels = 1 µm.

<u>DAPI Macro</u>

```
colour = "C3"
run("Set Scale...", "distance=6.236 known=1 pixel=1 unit=µm global");
run("Split Channels");
```

```
for (i = 1; i < (nImages+1); i++) {selectImage(i); title = getTitle(); if (indexOf(title, colour) != -1) {targetImage=getImageID();}}
selectImage(targetImage);
run("Subtract Background...", "rolling=10");
run("Duplicate...", "title=[workingImage]");
workingImage=getImageID();
setAutoThreshold("MaxEntropy dark");
run("Make Binary");
run("Convert to Mask");
run("Watershed");
run("Create Selection");
roiManager("Add");
roiManager("Split");
selectImage(targetImage);
roiManager("Show All");
roiManager("Measure");
```

<u>PI Macro</u>

```
colour = "C1"
run("Set Scale...", "distance=6.236 known=1 pixel=1 unit=µm global");
run("Split Channels");
for (i = 1; i < (nImages+1); i++) {selectImage(i); title = getTitle(); if (indexOf(title, colour) != -1) {targetImage=getImageID();}}
selectImage(targetImage);
run("Subtract Background...", "rolling=10");
run("Duplicate...", "title=[workingImage]");
workingImage=getImageID();
setAutoThreshold("MaxEntropy dark");
run("Make Binary");
run("Convert to Mask");
run("Watershed");
run("Create Selection");
roiManager("Add");
roiManager("Split");
selectImage(targetImage);
roiManager("Show All");
roiManager("Measure");
```

Supplemental References

S1. Siegel, T.N., Hekstra, D.R., and Cross, G.A.M. (2008). Analysis of the *Trypanosoma brucei* cell cycle by quantitative DAPI imaging. Mol. Biochem. Parasitol. *160*,171-174.

S2. Wheeler, R.J., Gull, K., and Gluenz, E. (2012). Detailed interrogation of trypanosome cell biology via differential organelle staining and automated image analysis. BMC Biology *10*.
